# Production of lignin based insoluble polymers (anionic hydrogels) by *C*. *versicolor*

**DOI:** 10.1038/s41598-017-17696-1

**Published:** 2017-12-13

**Authors:** Ivana Brzonova, Evguenii I. Kozliak, Anastasia A. Andrianova, Audrey LaVallie, Alena Kubátová, Yun Ji

**Affiliations:** 1 Department of Chemical Engineering, University of North Dakota, Grand Forks, North Dakota USA; 2 Department of Chemistry, University of North Dakota, Grand Forks, North Dakota USA

## Abstract

Unlike previous lignin biodegradation studies, white rot fungi were used to produce functional biopolymers from Kraft lignin. Lignin-based polymers (hydrogel precursors) partially soluble in both aqueous and organic solvents were produced employing a relatively fast (6 days) enzymation of Kraft lignin with basidiomycetes, primarily *Coriolus versicolor*, pre-grown on kenaf/lignin agar followed by either vacuum evaporation or acid precipitation. After drying followed by a treatment with alkaline water, this intermediate polymer became a pH-sensitive anionic hydrogel insoluble in either aqueous or organic solvents. The yield of this polymer increased from 20 to 72 wt% with the addition of 2% dimethylsulfoxide to distilled water used as a medium. The mechanical stability and buffering capacity of this hydrogel can be adjusted by washing the intermediate polymer/hydrogel precursor prior to drying with solvents of different polarity (water, methanol or ethanol). Any of these polymers featured a significant thermal resilience assessed as a high thermostable “coked” fraction in thermal carbon analysis, apparently resulting from significant covalent cross-linking that occurs during the treatment of their intermediate precursors.

## Introduction

Lignin, a large scale by-product of pulp and paper industry^1^ and bioethanol production^2^, is considered the most abundant source of renewable aromatics on Earth^3,4^. The current research efforts focus on production of phenolic monomers (e.g., vanillin, guaiacol) and other low-MW chemicals^5^, with their application in polymer industry^6^. The monomer production from lignin is usually low (5–35 wt%), with the rest being undesired phenolic oligomers or polymers^7,8^. Therefore polymers, either designed or undesired, are the primary ultimate products of lignin processing^3^.

Hydrogels represent a specific group of polymeric materials whose application is envisioned in many areas, e.g., in medicine as biomimetic scaffolds, wound healing materials or for drug delivery^9,10^, in food processing technology as thickening agents, stabilizers or food packaging materials^11^, and for waste water treatment^12,13^. Two main groups of hydrogels are permanent (chemical) and reversible (physical) hydrogels. In physical hydrogels the crosslinking involves hydrogen bonding, polyelectrolyte complexation, molecular entanglement, hydrophobic association and ionic bridging. All of these forces are relatively weak and therefore these hydrogels have a tendency to disintegrate over time^9,10^. By contrast, chemical hydrogels, which are made of rigid molecules like lignin, are crosslinked with covalent bonds thus being significantly more stable^14,15^.

Even though most of the currently used commercial hydrogels are based on synthetic polymers such as acrylic acid, poly(vinyl pyrrolidones) and poly(vinyl alcohols), some of them were recently produced from biobased polymers^16^. Starch and cellulose are already commonly used as a graft base whose properties can be adjusted with crosslinking density^17,18^.

Hydrogels have been obtained from various kinds of lignin such as Kraft lignin, lignosulfonates, and others^15,18^. Lignin based hydrogels are produced either through copolymerization with polyacrylamide, isopropyl acrylamide, N,N-methylenebisacrylamide, polyvinylalcohol, polyethylene glycol diglycidyl ether or cellulose, or by crosslinking with formaldehyde, glutaraldehyde, epichlorohydrin or epoxy compounds^15,18^.

This study presents an alternative, biological pathway to produce lignin-based hydrogels. To our best knowledge, no lignin-based hydrogels have been synthesized via biomodification. Recently, we described the production of a water-soluble lignin-based polymer^19^. Then, we discovered that this polymer can be converted into less soluble polymers; this information is reported in this article. However, the most significant finding reported here is an inexpensive (without the use of any crosslinking agents) and relatively fast conversion of these intermediate polymers into hydrogels of varied degree of functionalization, which are insoluble in various solvents, either aqueous or organic. As different hydrogels are formed when the intermediate polymer is washed with varied solvents, we provide their comparison and pertinent characterization, i.e., determination of swelling and buffering capacity and thermal resilience combined with elemental analyses of the resulting polymers.

## Results and Discussion

### Insoluble Polymers/Hydrogels (IP-H) production and its acceleration by dimethylsulfoxide (DMSO)

The insoluble lignin-based polymer/hydrogel, IP-H, was produced in three steps. First, the corresponding water-soluble polymer, APPL, was synthesized by cultivation of pre-grown and quasi-immobilized (on agar) fungi, primarily, *Coriolus versicolor*, as described in Section 4.2 of Materials and Methods. Enzymation^20^, i.e., uncoupling the culture growth and the subsequent feedstock treatment, was used conducting the latter in a solvent (distilled water, with or without DMSO) as opposed to an elaborate culture medium^19^.

Then, an IP-H precursor, intermediate polymer with a limited solubility in solvents was prepared by either vacuum evaporation or acid precipitation, as described in Section 4.3. Finally, an IP-H insoluble in both aqueous and organic solvents was produced by drying this intermediate product followed by its treatment in a highly alkaline solution and the final drying.

Table 1 shows the yields of both the soluble polymer (APPL) and insoluble IP-H for different fungal biotreatments. When lignin was not treated with fungi, small APPL and IP-H precursor amounts were still obtained but the insoluble hydrogel did not form at all (Table 1). Apparently, lignin oxidative cross-linking resulting from the fungal treatment is essential to render the polymer insoluble. As a result, the highly water soluble product of the fungal treatment (APPL)^19^ becomes highly insoluble during the dehydration process (drying) (Table 1).

**Table 1:** APPL and IP-H (washed in distilled water) yields as a result of 6-day fungal (*C*. *versicolor*) treatments with and without DMSO.

	Yield of APPL (wt. %)	Yield of IP-H (wt. %)
2% DMSO fungal treatment	84 ± 1	72 ± 1
2% DMSO control (no fungal treatment)	68 ± 2	0
0% DMSO fungal treatment	27 ± 1	20 ± 1
0% DMSO control (no fungal treatment)	8 ± 1	0

The IP-H yield was rather low while using water as a medium, without DMSO (Table 1). However, we discovered that the addition of 2% DMSO to this medium significantly increased the yield of not only APPL^19^ but also IP-H, from 20 to 72% (Table 1). Thus, DMSO as a solubilizing solvent is not essential for the IP-H formation but increases the lignin conversion as a result of fungal biotreatment. Elemental analysis (Section 2.4) showed that DMSO was not incorporated into the lignin being removed by washing with solvents.

Besides using DMSO, the key parameter in IP-H production was the composition of the agar blocks used for fungi quasi-immobilization and cultivation, prior to the Kraft lignin biological modification. The best results were obtained with the agar having a 1:1 ratio of lignin and lignocellulose (kenaf biomass) used as the growth medium for three generations. Either smaller or larger amounts of lignin resulted in a lower yield of IP-H. The absence of lignin in the agar cultivation/quasi-immobilization solid medium resulted in a near-zero IP-H yield as a result of subsequent liquid medium cultivation. Perhaps, the presence of an optimum lignin concentration in the agar growth medium enhances the induction of biosynthesis of specific enzymes. Another important parameter was the cultivation/quasi-immobilization time and storage of the strains prior to the Kraft lignin modification. The best results were obtained with 12 day old cultures followed by a short (12 to 24 h) storage in a fridge (~7 °C) prior to the lignin biomodification step.

### Intermediate polymer products obtained by acid precipitation or solvent evaporation

Based on the results presented in Table 2, a 6 day cultivation is sufficient for completion of a lignin treatment with *C*. *versicolor*. If the Kraft lignin modification with *C*. *versicolor* was performed for a longer time (12 days), the amount of solubilizable lignin remained virtually the same as on day 6.

**Table 2:** The fraction removed during the washing of IP-H precursors obtained by a 6-day incubation with *C*. *versicolor*.

Incubation time	Fraction of “solubilizable” lignin polymer removed (wt. %)		
(days)	Distilled water	Methanol	Ethanol
1	55 ± 7	100 ± 1	100 ± 1
2	54 ± 9	100 ± 1	100 ± 1
3	46 ± 3	100 ± 4	100 ± 1
4	32 ± 7	92 ± 7	100 ± 3
5	18 ± 5	50 ± 8	87 ± 10
**6**	**9 ± 3**	**21 ± 6**	**35 ± 9**
12	7 ± 3	20 ± 7	30 ± 5

Table 2 also reflects the gradual changes in lignin as a result of fungal treatment, i.e., the extent of polymer solubilization as a result of its washing with different solvents. The most apparent trend seen in Table 2 is a gradual shift (with time) of the washed polymer fraction towards a lower polarity as it is preferentially removed with less polar solvents. Yet, prior to precipitation the entire polymer product remained soluble in 2% aqueous DMSO thus showing its significant remaining polarity. Corroborating this statement, the precipitated polymer could still be re-dissolved in DMSO or in alkaline aqueous solutions (pH ≥ 9.5) either before or after washing with solvents, as long as it was not dried.

**Figure 1:**
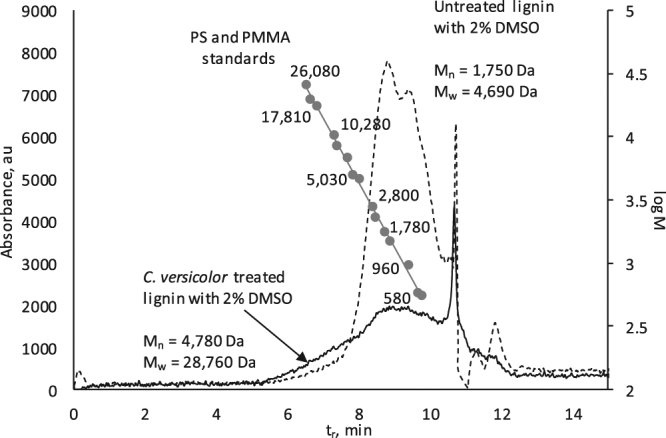
GPC profiles of untreated lignin and APPL obtained by a 6-day lignin treatment with *C. vercicolor,* along with the elution maxima of MW calibration standards whose MW values are shown on the graph.

An increase in lignin MW upon biomodification with *C*. *versicolor* was observed (Fig. 1). The modified lignin eluted from the GPC column earlier than the original lignin, thus evidencing the presence of higher MW species in the sample. Even though the absolute value of absorbance increased only slightly between 6–8 min of retention time, the impact of this *fraction* on the average MW is significant, as the abundance of low-MW fractions decreased concomitantly. The calculated number-average and weight-average MW values corroborated this observation showing an increase of MW_n_ from 1,750 Da to 4,780 Da and that of MW_w_ from 4,690 Da to 28,760 Da, respectively. This observation indicated that intermolecular cross-linking (presumably, oxidative cross-linking) is the major reaction path in lignin biomodification^19^.

The further processed APPL including both the hydrogels and their polymer precursors could not be studied by GPC or any other methods that require the sample to be completely dissolved, owing to their poor solubility in any solvents. Yet their lower solubility compared to APPL indicated their even higher extent of cross-linking and, presumably, MW.

Lignin crosslinking observed in this study has been known earlier, although it had not been identified as the dominant outcome of the fungal treatment. Oxidoreductases produced by basidiomycetes are usually linked to lignin degradation and decomposition towards monomers^8^. Yet, at the same time, those enzymes are also known to polymerize phenolic compounds (monomers and oligomers)^21^. The balance between lignin degradation and fragment polymerization/crosslinking depends on the reaction temperature, solvent addition, enzyme origin and on the feedstock structure^21^. Additional polymerization may occur due to the presence of carbohydrate impurities present in lignin, such as reactions of glycosidic hydroxyl groups, similar to oligomerization of rutin catalyzed by laccase from *T*. *versicolor* ^22^ or esculin polymerization^23^. Oxidative enzymatic crosslinking of phenolic biopolymers (isolated from *Fucus serratus*) was reported using vanadium-dependent bromoperoxidase and hydrogen peroxide^24^. Phenolic-functionalized macromolecules can also be enzymatically crosllinked by the phenolics present in those macromolecules, the observation most closely linked to the results obtained in our study^25^.

Corroborating these observations, a study performed by Prasetyo *et al*. using water soluble lignosulfonate and laccases in the presence of 1-hydroxybenzotriazole mediator showed up to 572% increase of lignin molecular weight. The polymerization resulted in the formation of additional ether and C–C aryl–aryl or aryl–alkyl linkages thus providing evidence for crosslinking^29^. These results are similar to those reported here. However, unlike our study, the lignosulfonate based polymer remained soluble after drying (as expected given the well-known high solubility of lignosulfonates).

In general, oxidoreductases promote both the decomposition reactions at the same time as those that form reactive intermediates, whose further processing can lead to crosslinking^26–28^. Perhaps, the enzymation conducted in this study in the absence of growth substrates, particularly with a DMSO addition, significantly tipped the balance between lignin depolymerization and cross-linking, so the latter became dominant.

After the biological modification, additional changes in the lignin structure were made *via* different separation and purification pathways. The intermediate polymer products (IP-H precursors) can be formed either by 1) immediate air drying (i.e., water evaporation from an APPL-containing solution), or 2) acid precipitation followed by washing with various solvents and air drying at 65 °C, as detailed in Section 4.3.

Precipitation (followed by washing with solvents) parenthetically removes DMSO as well as any soluble impurities, including the unreacted lignin. Based on the results presented in Table 2, the extent of mass reduction by applying different washing solvents correlates with Kraft lignin solubility in these solvents: 7% for water, 10% for methanol and 12% for ethanol (determined via gravimetric measurements, Section 4.8). It is of note that the solubility of Kraft lignin in nonpolar solvents (e.g., DCM) is near 0%, so these trends are consistent with its amphiphilic nature. The effects of washing with different solvents on resulting polymers’ physical and chemical properties are discussed in the following sections.

The variation of color between the precursors (shown in the Graphical Abstract) appears to be linked to their density rather than cross-linking. As the density of unmodified lignin was 412 mg/cm^3^, the low density (156 and 192 mg/cm^3^) precursors prepared by washing with methanol and ethanol respectively had lighter color compared to the rather dense (507 and 580 mg/cm^3^) precursors prepared by washing with water or vacuum evaporation, respectively.

### Preparation of insoluble polymers-hydrogels (IP-H)

The dried intermediate polymer precursors obtained as described in the previous section were treated with aqueous NaOH. Soaking such a polymer in aqueous solutions at an alkaline pH of 9–13 turned it into a swollen hydrogel. The swelling process was relatively slow, ~2 days. When the pH was returned to neutral, the polymer remained in the hydrogel form. However, when the pH was decreased to pH 3.5–1.5, the hydrogel shrunk back to its unhydrated form within seconds. Thus, the obtained product may be classified as an anionic hydrogel.

After the final drying step following the alkali treatment, the ultimate product became insoluble in any solvent. Thus, it will be called henceforth an “insoluble polymer-hydrogel,” IP-H, while the corresponding intermediate polymer products will be called IP-H precursors. The final yields of both this final product and its intermediate precursors washed by various solvents are listed in Table 3. The difference between the yields of IP-H and its precursor is due to the partial solubilization of the latter in aqueous NaOH when making the hydrogel. As expected, the alcohol-washed polymers did not lose much of their weight as a result of this treatment, as most of the unreacted lignin had been washed away. It was also expected that a sizable fraction of the *water-*washed IP-H precursor would be lost in this treatment, just as observed. As a result, the yields of all three IP-Hs obtained with the precursor polymer washing were similar.

**Table 3:** Yields of IP-H precursors and IP-H.

	**Yield %**	
	**IP-H precursor**	**IP-H**
**H**_**2**_ **O washed**	72 ± 1	54 ± 1
**MeOH washed**	64 ± 7	59 ± 2
**EtOH washed**	54 ± 5	49 ± 5
**Vacuum evaporation**	83 ± 3	**74 ± 3**

However, the observed much higher IP-H yield obtained by vacuum evaporation was not expected, as we originally assumed that washing with solvents removes only the unreacted lignin. Contrary to this assumption, only about 10% of this polymer dissolved in the aqueous NaOH solution while most of the fraction that would be removed by solvents became incorporated into the IP-H. Apparently, this fraction was significantly modified by fungal treatment though not as much as the rest of the IP-H.

The successful IP-H formation from its precursor obtained by vacuum evaporation shows that acid precipitation and solvent washing are not essential for IP-H formation whereas the drying step is essential. The necessity of drying, in turn, suggests that IP-H precursors undergo additional cross-linking during this procedure causing the chemical modification that defines the hydrogel properties. This suggestion was confirmed when studying the polymer properties, as detailed in the subsequent sections.

### Elemental composition of the polymers

The results of elemental analyses of both the IP-H obtained and their precursors are reported in Table 4. Notable is the observed consistent increase of the oxygen content of all dried hydrogels or xerogels as well as the IP-H precursor obtained by solvent evaporation. This observation indicates a greater extent of these polymers’ oxidation, which may partially involve crosslinking.

**Table 4:** Elemental analyses of lignin based polymers directly measuring the percentages of carbon, hydrogen, sulfur and nitrogen. The oxygen content was calculated as the difference between 100% and the sum of the percentages of measured elements.

Type of polymer	Carbon (%)	Hydrogen (%)	Sulfur (%)	Nitrogen (%)	Oxygen (%)*(calculated)*
Original Kraft lignin	63.52	5.68	1.38	0.68	28.74
IP-H precursor MeOH washed	60.51	5.54	4.81	0.44	28.7
IP-H precursor EtOH washed	60.66	5.65	4.41	0.33	28.95
IP-H precursor H_2_O washed	57.34	5.87	9.15	0.37	27.27
IP-H precursor vac. evap.	52.43	5.22	7.67	0.61	34.07
IP-H MeOH	63.82	5.06	0.91	0.55	29.66
Xerogel MeOH	56.26	5.47	0.91	0.65	36.71
IP-H vac. evap	62.93	5.22	1.02	0.67	30.16
Xerogel vac. evap	57.99	5.63	1.02	0.7	34.66
Xerogel EtOH	—	—	0.85	—	—

The other difference was observed in the sulfur percentage. The IP-H precursors showed a greater sulfur content, particularly, those obtained by solvent evaporation and water washing. Washing this polymer with alcohols partially removed sulfur, yet it remained larger than that in lignin. Presumably, the extra sulfur was introduced by DMSO.

However, the elemental analyses of hydrogels and xerogels indicated that crosslinking responsible for hydrogel formation was not due to the introduced sulfur. The resulting polymers showed lower amounts of sulfur than the IP-H precursors, similar to that of the original lignin. Apparently, the introduced sulfur was washed away by the alkaline solution applied when forming IP-H.

### Solubility of IP-Hs and their precursors

Whenever an IP-H preparation was unsuccessful, e.g., short incubation time, suboptimal inoculation conditions and other cases described in Section 2.1, the yields similar to those reported in Table 3 could be observed only for IP-H precursors whereas either zero or near-zero yields of the corresponding IP-Hs were recovered, as these precursors dissolved in the NaOH solution during the final step of the hydrogel preparation. Table 5 emphasizes this difference by showing the solubility of one of the IP-H precursors (the one washed with water) obtained through biomodification with 0 and 2% DMSO and compared to that of lignin. While the non-treated lignin dissolved in either several organic solvents or highly alkaline media, the IP-H precursor showed only rather limited solubility.

**Table 5:** Solubilization of the water washed IP-H precursor (obtained with a 6 day *C*. *versicolor* treatment) and original lignin (control).

**Solvents**	**Fraction of solubilized water-washed IP-H precursor (wt**. **%)**		**Fraction of solubilized water-washed IP-H Precursor (wt**. **%)**	
	**Lignin treated by fungi in 2% DMSO**	**Non-treated lignin (control) in 2% DMSO**	**Lignin treated by fungi in0% DMSO**	**Non-treated lignin (control) in 0% DMSO**
**DMSO**	15 ± 2	88 ± 5	12 ± 2	96 ± 3
**DMF**	9 ± 1	92 ± 2	11 ± 1	93 ± 3
**NMP**	9 ± 2	95 ± 1	10 ± 2	95 ± 3
**Dioxane**	0 ± 1	84 ± 2	0 ± 2	96 ± 2
**Ethylene glycol**	0 ± 1	81 ± 2	0 ± 1	90 ± 2
**Water pH 1**.**5**	0 ± 1	0 ± 1	0 ± 1	0 ± 1
**Water pH 7**	0 ± 1	3 ± 1	0 ± 1	5 ± 1
**Water pH 9**.**5**	3 ± 2	98 ± 3	5 ± 2	100 ± 3
**Water pH 13**	18 ± 3	100 ± 2	16 ± 3	100 ± 3

As the solubility of the IP-H precursor obtained with 0 and 2% DMSO was found to be statistically similar, we used henceforth only the samples obtained with 2% DMSO because of their much higher yield. Table 6 expands the solubility information to the rest of the obtained IP-H precursors, with the experimental details of solubility measurements shown in Section 4.5. The failure to obtain a hydrogel resulted in the IP-H complete or near-complete solubilization in alkaline water, just as for the control, untreated lignin, as shown in Table 5. Either long or repeated polymer precursor washing, particularly with alcohols, also increased its solubility in the NaOH solution thus lowering the hydrogel yield.

**Table 6:** Comparison of solubilization of IP-H precursors prepared from APPL washed with distilled water, methanol and ethanol and prepared via vacuum evaporation.

(wt% of solubilized IP-H precursors prepared with 2% DMSO)				
Solvents	IP-H precursor washed with distilled water	IP-H precursor washed with Methanol	IP-H precursor washed with ethanol	IP-H precursor obtained by vacuum evaporation
**DMSO**	15 ± 2	5 ± 1	7 ± 1	7 ± 1
**DMF**	9 ± 1	5 ± 1	7 ± 2	3 ± 1
**NMP**	9 ± 2	2 ± 1	4 ± 2	3 ± 1
**Dioxane**	0	0 ± 1	0 ± 1	0
**Ethylene glycol**	0	0	0	0
**Water pH 1**.**5**	0	0	0	0
**Water pH 7**	0	0	0	0
**Water pH 9**.**5**	3 ± 2	2 ± 1	3 ± 1	0 ± 1
**Water pH 13**	18 ± 3	5 ± 1	5 ± 1	9 ± 1

The successful IP-H precursors featured reduced solubility not just in alkaline water but also in organic solvents as shown in Tables 5 and 6, unlike the untreated lignin (Table 5). This observation corroborates the observed gradual reduction of the polymer solubility in all solvents as a result of successful biological modification shown in Table 2. The solubility in various organic solvents of the IP-H precursors produced with and without DMSO was statistically the same, thus confirming that this co-solvent did not affect the product but merely accelerated its formation.

As for the trends in solubility of IP-H precursors shown in Table 6, only DMSO and high-pH alkaline solutions removed some significant amounts of those polymers, yet these amounts were still small compared to the untreated lignin (Table 5). Most of the solubilization occurred during the first day, presumably when the unreacted and insufficiently modified lignin was washed away.

As shown above, washing the intermediate IP-H precursor with water and alcohols altered the solubility of the intermediate IP-H precursors and yields of the corresponding final products. We thus investigated whether these differences in IP-H processing could also affect the properties of the resulting polymer. The obtained results are discussed in the subsequent sections.

### The thermal resilience of lignin polymers

**Figure 2:**
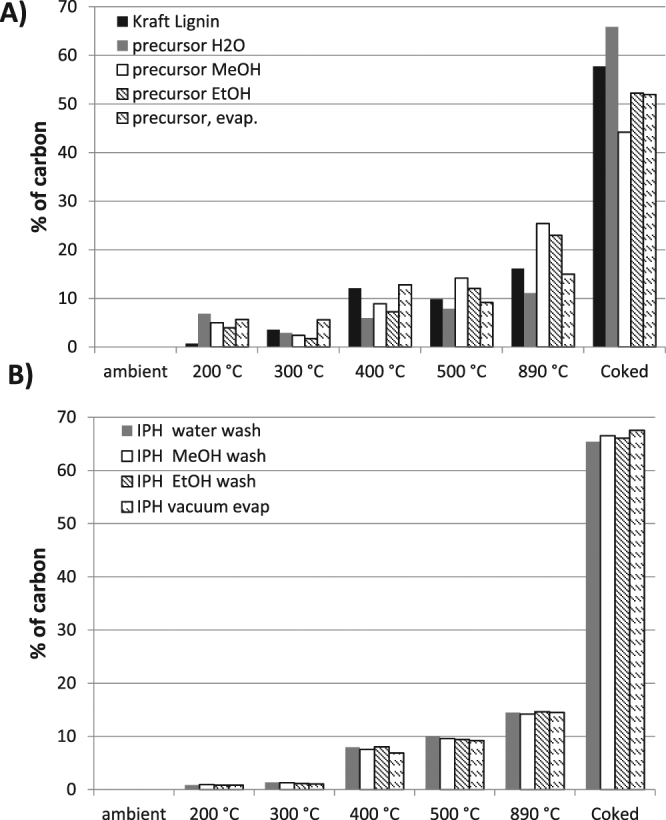
TCA temperature profiles of (**A**) IP-H precursors prepared from APPL washed with distilled water, methanol or ethanol and prepared via vacuum evaporation compared with original Kraft lignin and (**B**) IP-H after the final treatment with alkaline water and drying. (RSD < 5%).

The results of TCA analysis of the IP-H precursors produced in different protocols are shown in Fig. 2A, in comparison to both their earlier predecessor, a soluble polymer produced by lignin fungal treatment, and untreated lignin (used as a control). The most important information embodying thermal resilience is the amount of the last, “char” fraction that does not evolve at any temperature unless oxygen is added to burn it. The water-washed IP-H precursor showed an increase in this fraction compared to both the untreated and water-soluble lignin, as expected since some of the non-reacted or only slightly modified lignin was removed by washing. The char fraction appeared less pronounced in the non-washed IP-H precursor because the unreacted lignin was not yet removed from this polymer. It was less expected to observe an actual decline of this fraction in the alcohol washed IP-H precursors. Perhaps, alcohol washing removed a less polar and more cross-linked fraction of this polymer.

However, any differences in the abundance of the coked fraction disappeared when the hydrogels were formed from their polymer precursors (Fig. 2B). A significant increase in this recalcitrant fraction was observed, to near 80%. The increase in the coked fraction for the unwashed polymer can be explained by the removal of its less cross-linked part with the alkaline solution on the final step of its preparation. However, this explanation cannot be valid for alcohol-washed IP-H precursors, which did not lose much of their mass on the final step of preparation as shown in Tables 3 and 5. Apparently, the IP-H formation by the alkaline treatment followed by drying resulted in additional cross-linking, which corroborates the observed lack of IP-H solubility in both alkaline aqueous and organic solvents.

**Figure 3:**
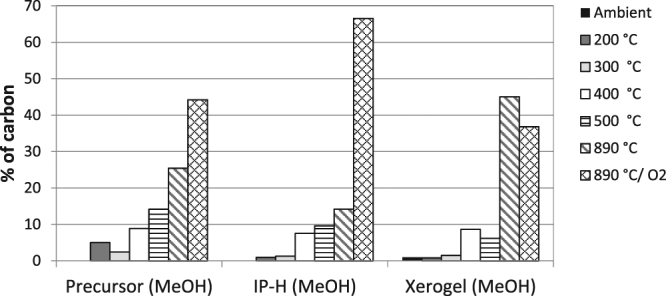
Comparison of TCA for an IP-H precursor (MeOH washed) with both the IP-H and xerogel prepared from this precursor. (RSD < 5%).

The colors of all xerogels become very light compared to those of the corresponding hydrogels (see the Graphical Abstract), which are rather dark. This significant difference cannot be explained solely by density and may indicate a lesser crosslinking in the former compared to the latter. Corroborating this hypothesis, the TCA results (Fig. 3) show a decrease in the char fraction in a xerogel compared to the corresponding hydrogel. Apparently, freeze drying when making the xerogel caused less crosslinking than thermal drying specific for the hydrogel preparation.

### Extent of swelling and response to changes in pH

**Figure 4:**
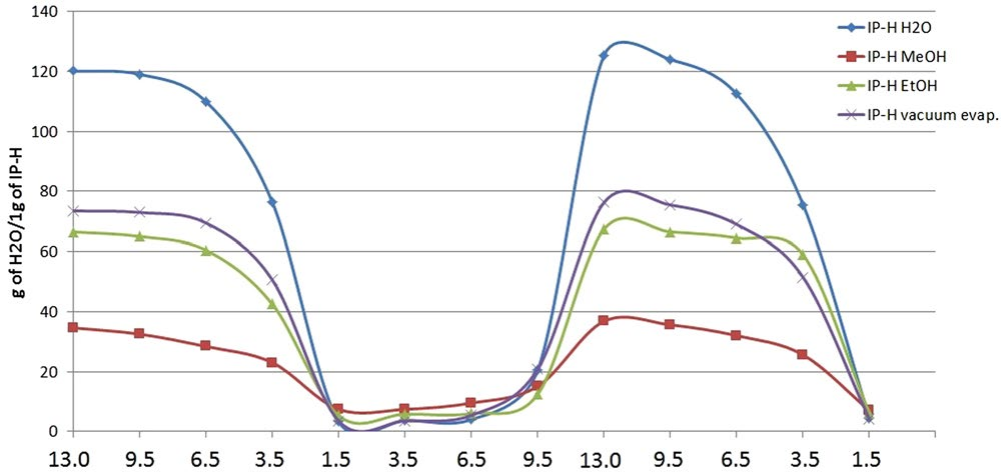
IP-H swelling response to pH changes.

Figure 4 shows IP-H swelling curves, i.e., responses in terms of water absorption/desorption to pH changes by adding either an acid (HCl, left and right) or base (NaOH, center). This profile is typical for hydrogels and indicates significant reversible swelling in alkaline media. The swelling occurs only as long as the weakly acidic functional groups remain deprotonated, so the IP-H may be characterized as an anionic hydrogel, as expected for a lignin based polymer.

A notable feature of the pH response shown in Fig. 4 is that the hydrogel swelling starts at a pH value as high as 9.5 and increases with the further NaOH addition; but when an acid is added, the gel remains 100% swollen at pH 9.5 and starts shrinking only near pH 7. This hysteresis can be explained by low accessibility of the bulk of deprotonated sites in the swollen gel, which can be overcome only by a large acid concentration causing a massive “unzipping” effect.

The pH response of this biotreated lignin polymer is similar to the behavior of some peptides^10,30^. Protein interactions with lignin were reported earlier^31^, so protein association with lignin might be viewed as a potential cause of IP-H formation. However, the TD-Pyr-GC-MS analysis conducted by us earlier for the water soluble polymer precursor^19^ showed that protein residues/markers were undetectable. In addition, the elemental analyses (Section 3.8) did not show any increase in nitrogen content for the biotreated samples. Thus, the hydrogel appears to be formed via the cross-linking of lignin phenolic units.

The IP-H swelling capacities calculated from the data of Fig. 4 are listed in Table 7. Most of the earlier reported lignin-based hydrogels featured swelling capacities around 10 g H_2_O/g gel but some of them absorbed up to 75 g H_2_O/g gel, e.g., the system of lignin - polyethylene glycol diglycidyl ether, where lignin was activated by O_2_ to improve crosslinking^18^. The biobased hydrogels reported here thus exhibit a reasonably strong swelling capacity. In particular, the water-washed IP-H precursor yields a hydrogel of a greater swelling capacity (~120 g H_2_O with 1 M NaOH/g gel) than those reported earlier while the alcohol-washed precursors produce the least swelling hydrogels.

**Table 7:** Swelling capacity of the obtained hydrogels.

**IP-H type**	**Swelling capacity** g of 1M NaOH/1 g gel
H_2_O washed	120 ± 13
MeOH washed	34 ± 4
EtOH washed	66 ± 5
Vacuum evap.	73 ± 5

As of note, the observed high swelling capacity of the water-washed hydrogel turned out to be detrimental to its mechanical stability. This hydrogel, unlike the other three, breaks apart even when simply transferred from one flask to another.

### Buffering capacity

**Figure 5:**
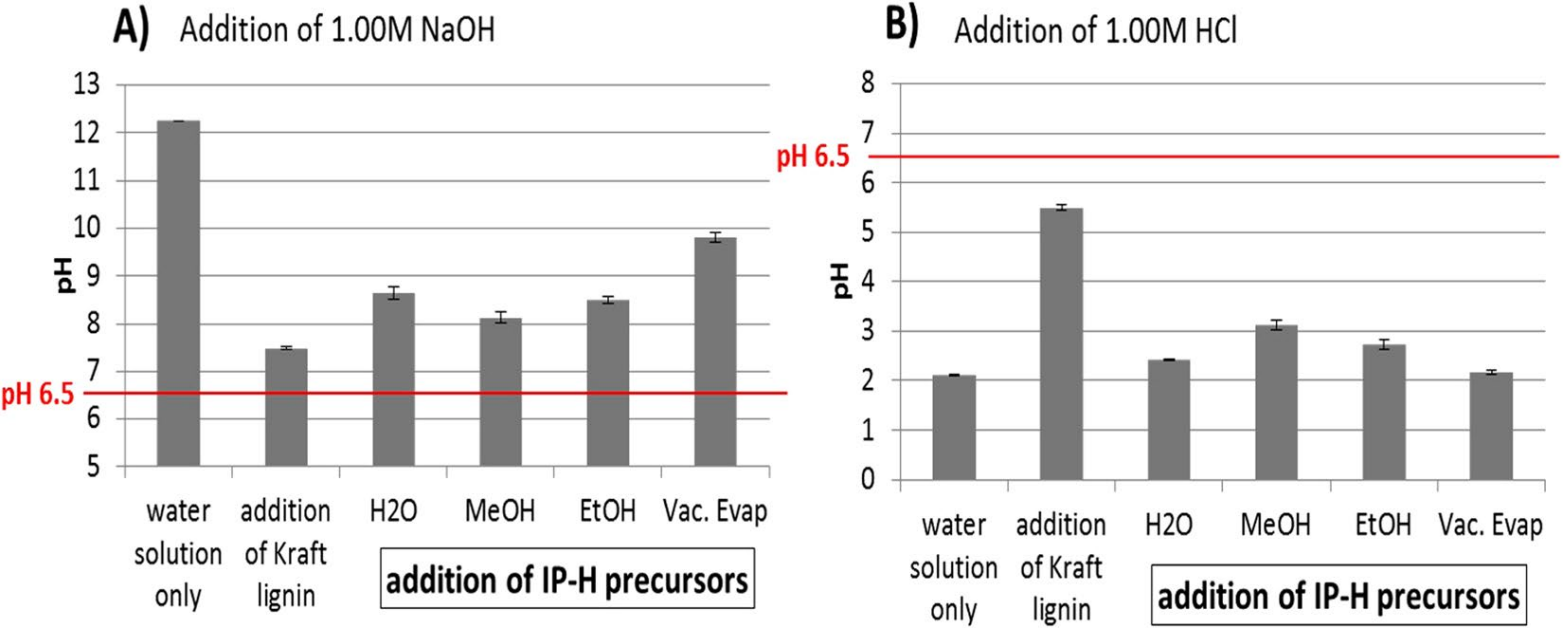
The effect of the preparation protocol, i.e., washing with solvents, on the “buffering” capacity of the polymers. (**A**) alkaline (**B**) acidic solutions.

The pH changes in response to either HCl or NaOH titration are shown in Fig. 5. For Kraft lignin, the buffer capacity was 50 mM for NaOH and 25 mM for HCl. This number was statistically similar for the water soluble APPL (data not shown).

The measurements of buffer capacity in the hydrogels turned out to be skewed. Namely, the buffer capacities with respect to NaOH addition became smaller than in the corresponding IP-H precursors while for HCl addition it became larger. Apparently, sizable amounts of NaOH were still stored within the hydrogels even at the solution pH = 6.5 or lower. This effect was less pronounced for the hydrogels with a greater swelling capacities, e.g., water-washed and prepared with vacuum evaporation, just as expected because the shrinking of swollen hydrogels should improve the release of the stored NaOH.

This observation suggests that the removal of functional groups occurs during the drying process, resulting in additional cross-linking. The decrease in buffering capacity continued with further washing the precipitated APPL with alcohols prior to drying. Apparently, washing with these solvents removed the most functionalized fraction of the polymer formed. The vacuum-dried IP-H precursor showed the smallest buffer capacity followed by the water-washed polymer. This trend is similar to that observed in TCA where the alcohol-washed samples showed a lower abundance of the coked fraction, cf. Fig. 2. Also, this trend correlates with the IP-H precursor yields (Table 3). It seems that more cross-linking (partially removing functional groups) occurs when the polymer is not washed with solvents.

### Morphological changes (SEM and confocal microscopy)

**Figure 6:**
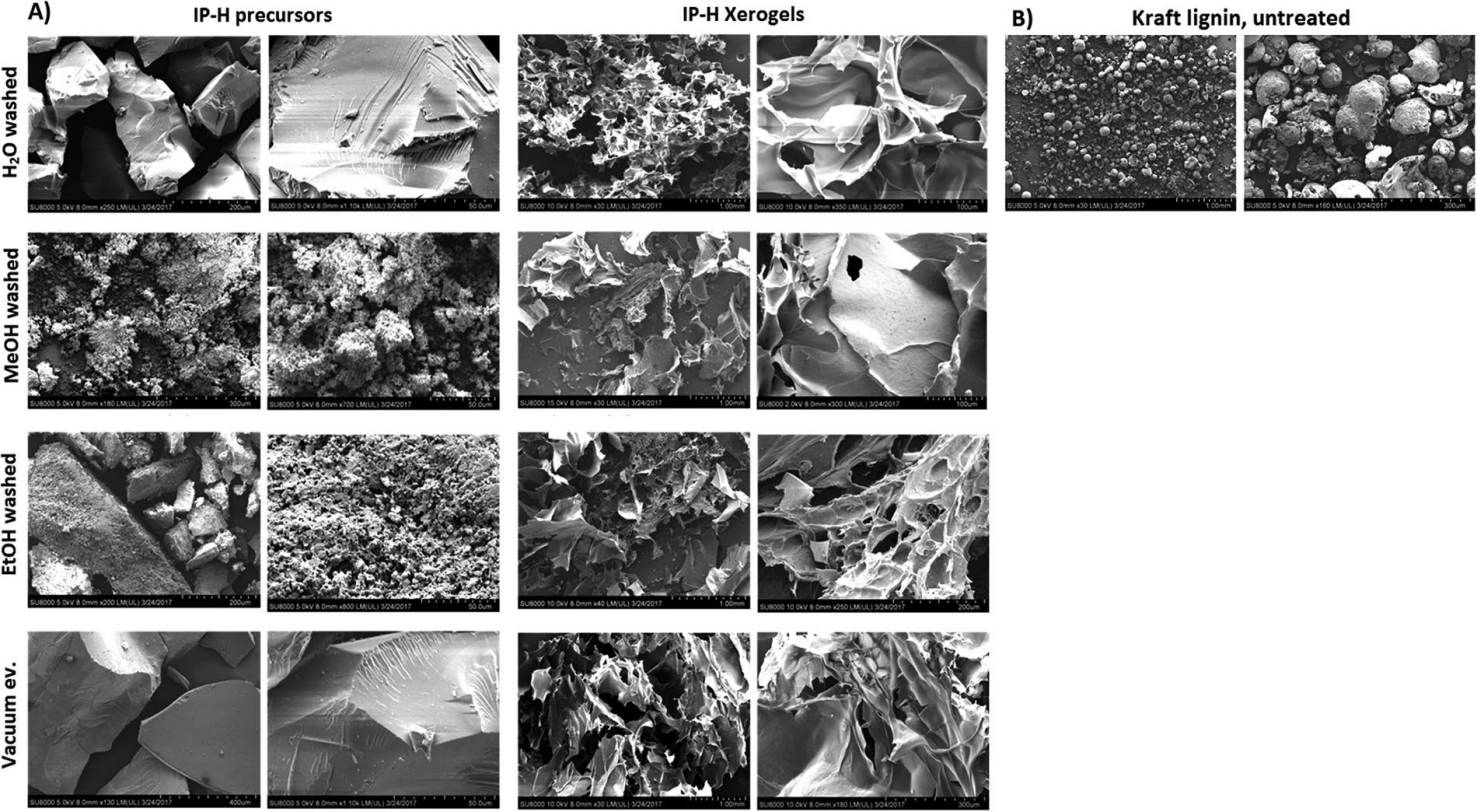
SEM of (**A**) the IP-H precursors and their corresponding xerogels, (**B**) the original Kraft lignin.

Based on SEM, the surface area of alcohol washed IP-H precursor appears to be high compared to the H_2_O washed IP-H or that obtained via vacuum evaporation (Fig. 6). The differences between the appearances of the corresponding xerogels (lyophilized hydrogels) are only minimal. Yet, the xerogel obtained from the hydrogel with the highest swelling capacity (prepared via H_2_O washing) appears to have a rather uniform pore size, with the walls having a similar thickness compared to the other xerogels. This uniformity might be owing to a more pronounced swelling expressed by this hydrogel, while the other hydrogels might not have reached their maximum swelling capacity before conversion into the corresponding xerogel, as swelling is a slow process.

### Potential IP-H applications

Broad applications of this bio-modified lignin polymer can be envisioned. One application may be waste water treatment in textile industries where the dyeing effluent exhibits high alkalinity (usually with pH between 9–13) and so these waters cannot be treated directly without a pH adjustment^12^. Another application of these lignin based hydrogels may be for water purification and desalination, where they can be used similar to modified graphene oxide membranes, or as sorbents for recalcitrant impurities, e.g., metals, prior to neutralization followed by a conventional treatment. Medical applications can also be envisioned where swelling under elevated pH can be applied in chronic non-healing wounds, which usually feature elevated pH, between 7 and 9^32^. Given the previously reported antioxidant, antimicrobial and anti-inflammatory properties of lignin^33^, the application of a lignin-based hydrogel can improve the healing of those wounds. This novel lignin based polymer has potential to replace/compete with lignin previously considered for various applications, such as ointment applications^34^, protein immobilization and potentially encapsulation^31^, imunosensing^35^, or as a functional/radiation protective coating^36^.

## Conclusion

**Figure 7:**
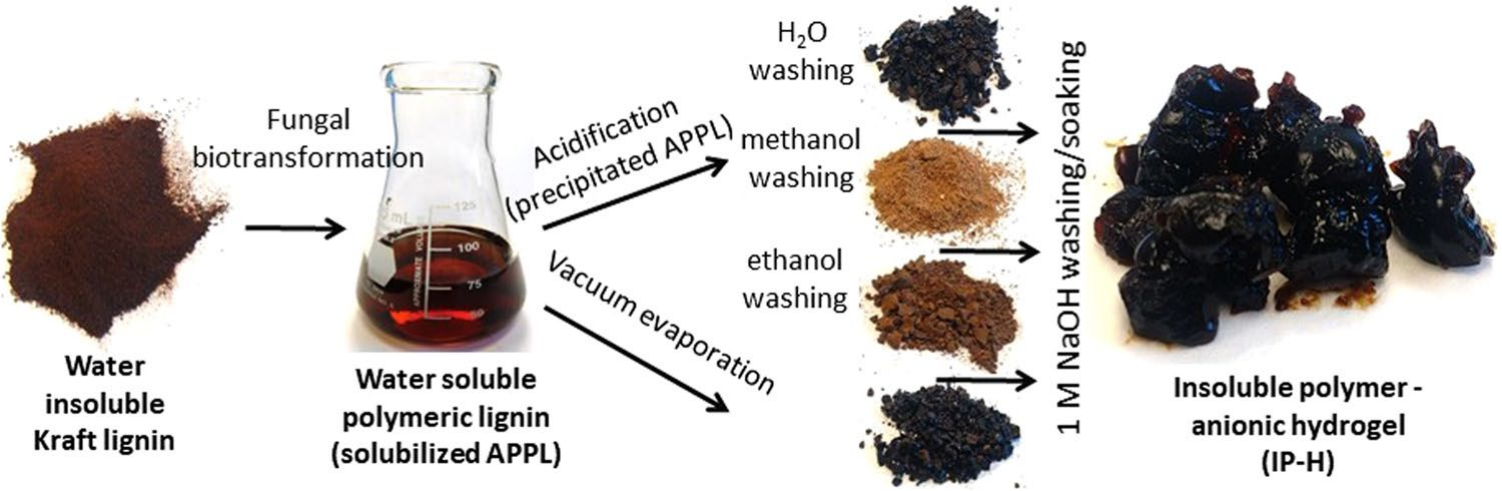
Graphical Abstract: Production of lignin based insoluble polymers (anionic hydrogels) by *C*. *versicolor*

A water-soluble polymeric product obtained by a 6-day lignin treatment in 2% DMSO with quasi-immobilized (pre-grown on agar) *Coriolus versicolor*, was converted into another polymer product featuring a limited solubility in both aqueous and non-aqueous solvents. By treatment with alkaline water followed by air drying, this polymer was transformed into a hydrogel, which is insoluble in either pure organic solvents (DMSO, DMF, NMP, dioxane, ethylene glycol), alcohols or aqueous media at pH 1.5–13.0. The yield, extent of cross-linking, swelling and buffer capacities, and functionalization of this polymer can be varied by an optional acid precipitation, followed by washing the acid precipitated product with different solvents. Yet the greatest yield of this polymer, with the highest buffer capacity and a suitable stability, can be obtained without any precipitation or washing. This hydrogel has potential for environmental and medical applications Fig. 7.

## Materials and Methods

### Feedstock, chemicals and solvents

Kraft lignin (alkali lignin) and all other chemicals except for solvents were purchased from Sigma Aldrich, St. Louis, MO, USA. The non-stabilized (i.e., those without additional chemicals) solvents used, including DMSO, tetrahydrofuran (THF), dioxane and ethylene glycol of either HPLC or spectrophotometric grade, were obtained from VWR International, Radnor, PA, USA.

### Fungal strains used

Biological treatment of Kraft lignin was performed with five fungal strains (*C*. *versicolor*, *T*. *galica*, *P*. *ostreatus*, *P*. *pulmonarius and G*. *lucidum*), with only small differences observed in performance between these strains. Therefore, we present only the results obtained with *C*. *versicolor*, which consistently produced the highest yield of the lignin based insoluble polymer/hydrogel (IP-H). Results and comparison of those strains are mentioned in our previous work^19^.

### Modification of insoluble Kraft lignin into a soluble polymer

This modification is described in great detail in our previous publication^19^ including a detailed chemical characterization of the resulting biomodified lignin. Below we provide a shorter description; note that in this study a ten-fold higher lignin concentration was used. Prior to inoculation of the liquid cultivation flasks, fungi (*Coriolus versicolor*) were pre-grown on agar. The agar medium used (300 mL) contained 3.0 g of Kraft lignin, 3.0 g of dry kenaf grass (*Hibiscus cannabinus*) and 4.5 g of agar. This medium, after sterilization in an autoclave (121 °C/ 30 min), was poured on Petri dishes. The solid agar was inoculated with 5 × 5 mm fragments of fully grown fungal cultures. The plates were incubated for 12 days at 22 °C.

These quasi-immobilized fungi were used for biomodification of the lignin containing liquid media. For samples without DMSO, 2 g of Kraft lignin was transferred into an Erlenmeyer flask together with 100 mL of distilled water. For DMSO containing samples, 2 g of Kraft lignin was first solubilized in 2.0 vol% of DMSO and then distilled water was added. The agar with fungi was cut into small fragments (ca. 5 × 5 mm), with approximately ¼ of the agar plate used for incubation of one Erlenmeyer flask. The Erlenmeyer flasks were placed in a shaker with the temperature set up at 29 °C for 6 days. Experiments were conducted in triplicate.

### Production of intermediate polymers, hydrogel precursors

**Figure 8:**
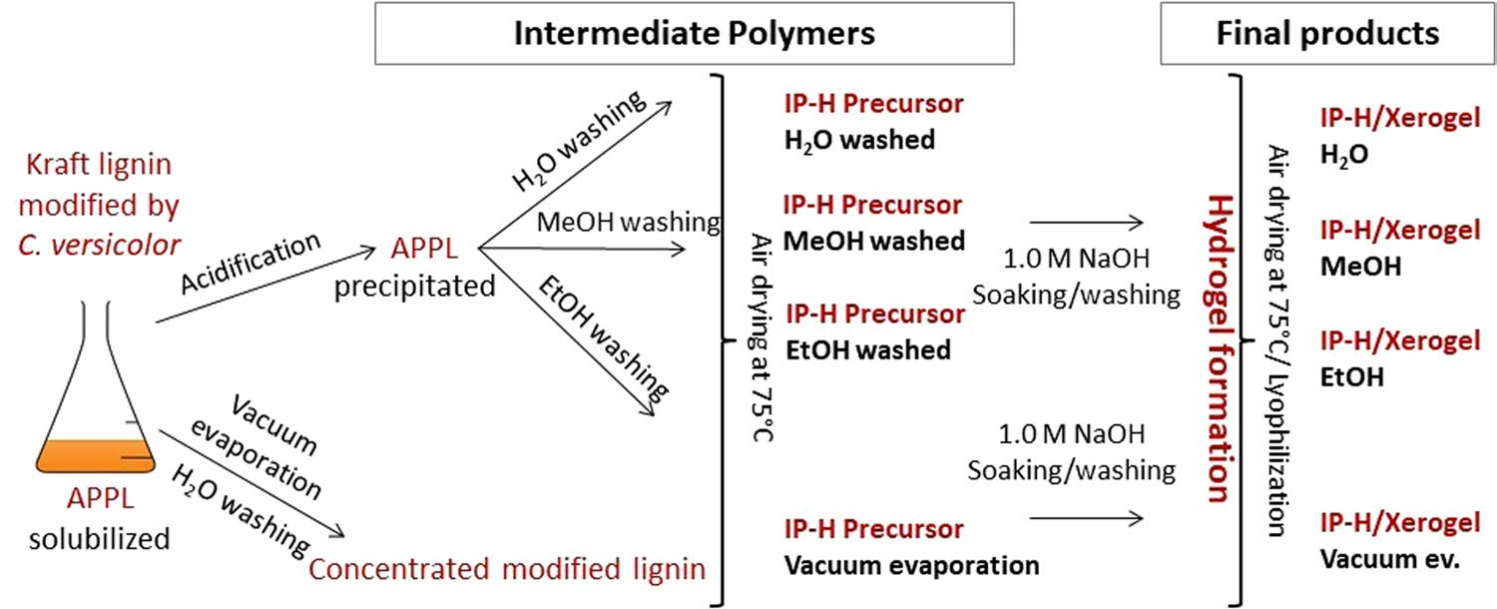
Experimental setup.

The water-soluble polymer prepared as described in the previous section was converted into the corresponding less soluble polymers using 100 mL of the reaction medium. Two separate methods were applied (Fig. 8):

1. Acidification to pH ~3.5 with the addition of 1 M HCl. This treatment caused the precipitation of acid precipitable polymeric lignin (APPL). To collect the APPL, samples were centrifuged (at 5,000 rpm for 15 min) and the sediment was collected. To remove the remaining DMSO or any other impurities, the samples were washed three times with 100 mL of either distilled water, ethanol or methanol. After washing, the precipitated APPL was dried at 75 °C for 48 hours (until a constant weight). The water washes of precipitated APPL could be done repeatedly without affecting the subsequent hydrogel formation. By contrast, washing with alcohols was done briefly and only once because long or repeated washing was shown to inhibit the subsequent hydrogel formation by solubilizing the precipitated APPL. Then, the solid was transferred to an oven and dried to a constant weight at 75 °C. Based on elemental analyses (Section 2.4), this washing step was able to remove most of the DMSO (mainly ethanol and methanol washing). The remaining DMSO was removed during the soaking phase of the hydrogel formation (including those IP-H precursors that were not washed), so no DMSO was present in the final product.
2. Vacuum evaporation method. The hydrogel precursor was prepared without precipitation by acidification. In this case, DMSO had to be removed to form a solid polymer. For this purpose, 100 mL of the water soluble lignin polymer solution (10 g/L) were evaporated down to ~5 mL using vacuum rotary evaporation (70–75 °C/25 in Hg). Then another 100 mL of distilled water were added and evaporated. This step was repeated 3 times to remove the bulk of DMSO. The last step continued until the liquid evaporated completely. The solid was then transferred to an oven and dried to a constant weight at 75 °C.

The main purpose of using vacuum evaporation was to prove that the gel formation/increased crosslinking is not connected to the precipitation by acidification.

### Production of insoluble polymer/hydrogel (IP-H)

The hydrogels were formed by the final treatment of their precursors with an aqueous 1.0 M NaOH solution. Approximately 1 gram of the gel was soaked in 100 mL of 1.0 M NaOH for 48 hours. After this treatment, the hydrogels were returned to their acidic forms by adding 0.1 M HCl and washed with distilled water. For gravimetric measurements, they were dried to a constant weight.

### Xerogel preparation

Hydrogels were prepared as described in Section 4.4. Approximately 1 g of the gel was soaked in 100 mL of distilled water for 48 hours to remove excessive NaOH and then the hydrogel was transferred into 2 mL Eppendorf tubes. In order to create a xerogel, this gel was frozen followed by drying via lyophilization.

### GPC analysis

#### Instrumentation

GPC analyses were performed on an Agilent 1100 Series HPLC with DAD detection (Agilent Technologies, Santa Clara, CA, USA). A PLgel 1000 Å (5 µm, 7.5 mm × 300 mm, 1000 Å) with a guard column (7.5 mm × 50 mm) (Agilent) was utilized. Unstabilized THF was used as a mobile phase at a flow rate of 1.0 mL/min. Polystyrene standards with M_p_ 580–19,760 Da purchased from Varian (Amherst, MA, USA) and poly(methyl methacrylate) standards with M_p_ 550–26,080 Da purchased from Agilent Technologies were used for the GPC column calibration. To prepare samples for GPC analysis, an APPL filtrate was dissolved in the THF-water system at a ratio of 1:1 v/v as a 1.0 mg mL^−1^ concentrate. The concentration of standards and lignin was 0.1 and 1.0% dissolved in THF and THF-water 1:1 v/v, with injection volumes 100 µL and 50 µL, respectively.

#### Data Handling

To eliminate DMSO and water artifacts from the chromatograms, a blank sample consisting of THF-water-DMSO 49–49–2% (v/v) was analyzed. Its chromatogram was subtracted from the chromatograms of the samples prior to molecular weight (MW) calculation. To calculate the number average (M_n_) and weight average (M_w_) MW of lignin samples, the total absorbance (in au) at the wavelength range between 220–750 nm (*A*_*i*_) was used. The absorbance at measurement point *i* had to exceed the baseline noise at least 3 times to be considered an analytical signal. The number-average and mass-average MW values, M_n_ and M_w_, respectively, were calculated in MS Excel using the standard formulas 1 and 2:

1 $${M}_{n}=\,\frac{{\sum }^{}{A}_{i}{M}_{i}}{{\sum }^{}{A}_{i}}$$

2 $${M}_{w}=\,\frac{{\sum }^{}{A}_{i}{M}_{i}^{2}}{{\sum }^{}{A}_{i}{M}_{i}}$$

To calculate sample’s effective MW (*M*_*i*_) at measurement point *i*, a linear calibration was used based on the standard MW plotted vs. retention time, with the corresponding data provided within the same plot (Fig. 1).

### Measurement of polymer solubility in solvents

The intermediate lignin polymer products (Section 4.3) as well as the original lignin and final products, IP-Hs (Section 4.4), were subjected to a solubility evaluation test. Dried samples were divided into small, ~1 mm, pieces and 1.00 g of each sample was subjected to solubilization in 10 mL of either an organic solvent (DMSO, THF, dioxane or ethylene glycol) or aqueous solution at pH 1.5, 7, 9 and 13. 20-mL glass vials containing 1.00 g of this polymer (biologically treated dried lignin) in 10.0 mL of a solvent were placed in a shaker incubator at 21 °C. Solubilization was conducted in three 48 h steps followed by centrifugation. After centrifugation, the supernatant was removed and replaced with a fresh solvent, this step was repeated three times followed by the sample drying at 75 °C to a constant weight.

### Hydrogel swelling capacity measurements in response to a pH change and a cursory assessment of mechanical stability

To determine the swelling capacity of the biologically modified lignin polymers, 1.00 g of a polymer was placed into 100.0 mL of 1.0 M NaOH for 72 hours, which was sufficient to convert the insoluble lignin polymer into the corresponding hydrogel. After 72 hours, the swollen hydrogel was placed for one hour on filter paper to remove excessive water, then the hydrogel was weighed. The swelling capacity was calculated as the amount of water absorbed by 1 g of the original polymer.

The pH response was evaluated as follows. First, when 72 hours of swelling in 1.0 M NaOH were applied to obtain a hydrogel, the amount of water in 1 g of this polymer was measured. This hydrogel was placed into an Erlenmeyer flask containing 100 mL of a 1.0 M NaOH aqueous solution at pH 9.5 for 24 hours. The hydrogel was taken out, weighed, then submerged in 100 mL of distilled water (pH 6.5) for 24 hours and weighed again. The water content was determined as the weight difference. This procedure was repeated at pH 3.5 and then at pH 1.5, with the measurement of the polymer water content after each step. Then these steps were repeated in the opposite order to check if the hydrogel would swell again.

### Fractionation by thermal evolution: TCA analysis

To evaluate the thermal resilience of the biotreated polymers in comparison to the original Kraft lignin, thermal carbon analysis (TCA) was used. The TCA was performed on an organic carbon/elemental carbon analyzer from Sunset Laboratory Inc. (WA, USA). The method used was previously reported by Asina *et al.* ^37^. The DMSO removal procedure by repeated evaporation with water described in Section 4.3 was used for TCA sample preparation to ensure their low percentage of sulfur, which is detrimental for the instruments optics.

The TCA thermal evolution profiles were obtained at 200, 300, 400, 500 and 870 °C under an inert helium atmosphere followed by one additional step conducted at 850 °C with oxygen, which allowed for quantification of the non-volatile char portion in samples. TCA thus generates results similar to those of thermogravimetric analysis, TGA, but also allows for a mass balance closure on carbon by accounting for the non-volatilizable “coked” fraction. The TCA sample preparation required the removal of DMSO: The corresponding procedure is described in Section 4.3, as it was also used for preparation of the vacuum-evaporated IP-H precursor. All samples were analyzed in duplicate with the relative standard deviation (RSD) being lower than 5%.

### Polymer buffering capacity determination

Erlenmeyer flasks containing 25 mL of distilled water were supplemented with 0, 100, 200, 300, 400, 500 and 1000 µL of 1 M NaOH (or 1 M HCl). 1.0 g of either untreated lignin or a biologically modified lignin polymer was added to each of these flasks. A similar sample without lignin was used as a blank. These flasks containing an alkali/acid medium and lignin were placed into an incubator shaker for 1 h. After the incubation, the pH was measured. The buffering capacity was calculated as the number of moles of either a strong base or acid required to change the pH of 6.5 in 100 g/L (lignin polymer in water) by one unit. The pH 6.5 is that native to aqueous solutions with Kraft lignin or to IP-H precursors.

To determine the hydrogel buffering capacity, 1 g of a certain IP-H precursor was soaked and washed in 1.00 M NaOH for 48 hours to obtain the corresponding hydrogel. Then the hydrogel was transferred in 100 mL of aqueous solution with HCl (pH 4.5) for 1 hour (the pH of the solution increased to ~12 because the NaOH inside the hydrogel) then the liquid was removed and replaced with new HCl solution (pH 4.5). This step was repeated until the pH of the solution was around 7.5. Then continuing washing was done with distilled water only (~pH 6.5). When the solution pH stabilized ~6.5 and then remained stable for 24 hours of soaking, either NaOH or HCl were added to observe the buffering capacity. Namely, either 500 µL of 1.00 M NaOH or 250 µL of 1.00 HCl were added to the hydrogels in aqueous solutions at pH 6.5, then the samples were incubated at room temperature for 24 hours and the pH was measured after this incubation to determine the hydrogel ability to stabilize pH.

### SEM analysis

Dried IP-H precursors and xerogels were attached on a carbon tape and coated with carbon to increase conductivity, followed by examination under a field emission scanning electron microscope, SEM (Hitachi, SU8010, Japan).

## References

[1] Gellerstedt G (2015). Softwood kraft lignin: Raw material for the future. Ind. Crops Prod..

[2] Doherty WOS, Payam M, Fellows CM (2011). Value-adding to cellulosic ethanol: Lignin polymers. Ind. Crops Prod..

[3] Fang, Z. & Smith, Z. L. Production of Biofuels and Chemicals from Lignin Biofuels and Springer (2016).

[4] Dai J, Patti AF, Saito K (2016). Recent developments in chemical degradation of lignin: catalytic oxidation and ionic liquids. Tetrahedron Lett..

[5] Davis KM (2016). Recovery and utilization of lignin monomers as part of the biorefinery approach. Energies.

[6] Upton BM, Kasko AM (2015). Strategies for the conversion of lignin to high-value polymeric materials: Review and perspective. Chem. Rev..

[7] Kozliak EI (2016). Thermal liquefaction of lignin to aromatics: efficiency, selectivity, and product analysis. ACS Sustain. Chem. Eng..

[8] Asina F, Brzonova I, Kozliak E, Kubátová A, Ji Y (2017). Microbial treatment of industrial lignin: Successes, problems and challenges. Renew. Sustain. Energy Rev..

[9] Aguilar MR, Elvira C, Gallardo A, Vázquez B, Román JS (2007). Smart polymers and their applications as biomaterials. J. Tissue Eng..

[10] Ago M, Okajima K, Jakes JE, Park S, Rojas OJ (2012). Lignin-based electrospun nanofibers reinforced with cellulose nanocrystals. Biomacromolecules..

[11] Shen X, Berton P, Shamshina J, Rogers RD (2016). Preparation and comparison of bulk and membrane hydrogels based on Kraft and ionic liquid isolated lignins. Green Chem..

[12] Shen C, Shen Y, Wen Y, Wang H, Liu W (2011). Fast and highly efficient removal of dyes under alkaline conditions using magnetic chitosan-Fe(III) hydrogel. Water Res..

[13] Yao Q, Xie J, Liu J, Kang H, Liu Y (2014). Adsorption of lead ions using a modified lignin hydrogel. J. Polym. Res..

[14] Gerlach, G. & Arndt, K. F. Hydrogel Sensors and Actuators. Springer (2010).

[15] Passauer L (2012). Highly swellable lignin hydrogels: Novel materials with interesting properties. ACS Symp. Ser..

[16] Nagam SP, Jyothi AN, Poojitha J, Aruna S, Nadendla RR (2016). A comprehensive review on hydrogels. Int. J. Curr. Pharm. Res..

[17] Shen X, Shamshina JL, Berton P, Gurau G, Rogers RD (2015). Hydrogels based on cellulose and chitin: fabrication, properties, and applications. Green Chem..

[18] Thakur VK, Thakur MK (2015). Recent advances in green hydrogels from lignin: A review. Int. J. Biol. Macromol..

[19] Brzonova I (2017). Fungal biotransformation of insoluble kraft lignin into a water soluble polymer. Ind. Eng. Chem. Res..

[20] Faber, K. Biotransformations in Organic Chemistry. Springer (2011).

[21] Ghoul, M. & Chebil, L. Enzymatic Polymerization of Phenolic Compounds by Oxidoreductases. Springer (2016).

[22] Anthoni J (2008). Investigation of enzymatic oligomerization of rutin. Rasayan J. Chem..

[23] Muñiz-Mouro A (2017). Comprehensive investigation of the enzymatic oligomerization of esculin by laccase in ethanol: water mixtures. RSC Adv..

[24] Berglin M, Delage L, Potin P, Vilter H, Elwing H (2004). Enzymatic cross-linking of a phenolic polymer extracted from the marine alga Fucus serratus. Biomacromolecules.

[25] Roberts JJ, Naudiyal P, Lim KS, Poole-Warren LA, Martens PJ (2016). A comparative study of enzyme initiators for crosslinking phenol-functionalized hydrogels for cell encapsulation. Biomater. Res..

[26] Leutbecher H (2011). Laccase-catalyzed phenol oxidation. Rapid assignment of ring-proton deficient polycyclic benzofuran regioisomers by experimental 1H-13C long-range coupling constants and DFT-predicted product formation. Org. Biomol. Chem..

[27] Chen M (2017). Radical mechanism of laccase-catalyzed catechol ring-opening. Wuli Huaxue Xuebao/ Acta Phys. - Chim. Sin..

[28] Mazloum S, Al-Ansari MM, Taylor K, Bewtra JK, Biswas N (2016). Additive Effect on Soybean Peroxidase-Catalyzed Removal of Anilines from Water. Environ. Eng. Sci..

[29] Prasetyo EN (2010). Polymerization of lignosulfonates by the laccase-HBT (1-hydroxybenzotriazole) system improves dispersibility. Bioresour. Technol..

[30] Shen W, Lammertink RGH, Sakata JK, Kornfield JA, Tirrell DA (2005). Assembly of an artificial protein hydrogel through leucine zipper aggregation and bisulfide bond formation. Macromolecules.

[31] Yamaguchi A, Isozaki K, Nakamura M, Takaya H, Watanabe T (2016). Discovery of 12-mer peptides that bind to wood lignin. Sci. Rep..

[32] Gethin G (2007). The significance of surface pH in chronic wounds. Wounds UK.

[33] Sakagami H (2005). Molecular requirements of lignin-carbohydrate complexes for expression of unique biological activities. Phytochemistry.

[34] Mahata D (2017). Lignin-graſt-polyoxazolin conjugated triazole a novel anti- infective ointment to control persistent inflammation. Sci. Rep..

[35] Cerrutti, B. M., Moraes, M. L., Pulcinelli, S. H. & Santilli, C. V. Lignin as immobilization matrix for HIV p17 peptide used in immunosensing. *Biosens*. *Bioelectron*. **71**, 420–426 (2015). 10.1016/j.bios.2015.04.054 25950938

[36] Shankar S, Rhim JW (2017). Preparation and characterization of agar/lignin/silver nanoparticles composite films with ultraviolet light barrier and antibacterial properties. Food Hydrocoll..

[37] Voeller, K. M. Characterization of kraft alkali lignin and producst of its thermal degradation by fractional pyrolysis. Master Thesis, Analytical Chemistry, University of North Dakota (2016).

